# The roles of symmetry and elongation in developing reference frames

**DOI:** 10.3389/fpsyg.2024.1402156

**Published:** 2024-07-01

**Authors:** Dongcheng He, Haluk Ogmen

**Affiliations:** ^1^Department of Electrical and Computer Engineering, Ritchie School of Engineering and Computer Science, University of Denver, Denver, CO, United States; ^2^Herbert Wertheim School of Optometry and Vision Science, University of California, Berkeley, Berkeley, CA, United States

**Keywords:** reference frame, object recognition, mental rotation, sensorimotor, learning

## Abstract

Previous studies showed that elongation and symmetry (two ubiquitous aspects of natural stimuli) are important attributes in object perception and recognition, which in turn suggests that these geometrical factors may contribute to the selection of perceptual reference-frames. However, whether and how these attributes guide the selection of reference-frames is still poorly understood. The goal of this study was to examine systematically the roles of elongation and symmetry, as well as their combination, in the selection of reference axis and how these axes are developed for unfamiliar objects. We designed our experiments to eliminate two potential confounding factors: (i) extraneous environmental cues, such as edges of the screen, etc. (by using VR) and (ii) pre-learned cues for familiar objects and shapes (by using reinforcement learning of novel shapes). We used algorithmically generated textures with different orientations having specified levels of symmetry and elongation as the stimuli. In each trial, we presented only one stimulus and asked observers to report if the stimulus was in its original form or a flipped (mirror-image) one. Feedback was provided at the end of each trial. Based on previous studies on mental rotation, we hypothesized that the selection of a reference-frame defined by symmetry and/or elongation would be revealed by a linear relationship between reaction-times and the angular-deviation from either the most symmetrical or the most elongated orientation. Our results are consistent with this hypothesis. We found that subjects performed mental rotation to transform images to their reference axes and used the most symmetrical or elongated orientation as the reference axis when only one factor was presented, and they used a “winner-take-all” strategy when both factors were presented, with elongation being more dominant than symmetry. We discuss theoretical implications of these findings, in particular in the context of “canonical sensorimotor theory.”

## 1 Introduction

In physics, reference-frames are crucial in analyzing and expressing the physical properties and relationships of objects in the environment. Likewise, our visual system relies extensively on reference-frames in processing visual information to carry out daily tasks, such as object recognition, motion detection, spatial reasoning, etc. (Rock, 1973; Palmer, 1975; Morvan and Wexler, 2005; Knapen et al., 2009; Agaoglu et al., 2015; He and Öğmen, 2023). Some reference-frames that our brain uses are encoded directly in our neural system. For example, our visual system begins with two retinas formalizing retinotopic maps, and this retinotopic reference-frame is maintained in the primary visual cortex (V1) (Fox et al., 1987; Engel et al., 1997). On the one hand, while the retinotopic reference-frame is egocentric as it is relative to the body, our visual system also uses exocentric reference-frames, which are with respect to a reference outside our body. For object recognition tasks, objects in the environment often appear in various ways, such as different sizes, orientations, or viewpoints. Many studies suggested that, to be stored and recognized, objects need to be represented based on their exocentric reference-frames and transformations were carried out to compensate the difference between the stored representation and a given visual input (Palmer et al., 1981; Tarr et al., 1998; Kourtzi and Shiffrar, 1999; Fang and He, 2005). On the other hand, not only can reference-frames be innately encoded (like the retinotopic maps in V1), but they can also be influenced through active cognitive development or learning. For example, perceptual learning was found to be effective in generating non-retinotopic reference-frames (Otto et al., 2010; Zhang and Li, 2010).

Mental rotation has been proposed as an active transformation mechanism to compensate for rotational variances in stimulus appearance. This is in line with sensorimotor theories of intelligence. For example, the first stage in the Piagetian theory of cognitive development is the sensorimotor stage. This is the stage underlying the emergence of object concept and constancy. Starting with innate reflexes, such as sucking, infants gradually build a repertoire of sensorimotor schema which are then “internalized” in the sense that the sensorimotor schema do not have to be executed physically but can be “simulated” mentally. Through this internalization, the infant does not need to grope or experiment physically by motor action, but can solve problems through “mental combination” (Piaget, 1952). Mental combination involves internal simulation of sensorimotor schema. Whereas Piaget built his theory mainly through behavioral observations, more recent neurophysiological studies provide support for the internalized sensorimotor schema. Previous studies have found evidence showing sensorimotor strategies in object recognition tasks. For example, one study reported correlations between sensorimotor networks and facial expression recognition (Wood et al., 2016). In another study, it was argued that subjects with multimodal agnosia, a visual recognition deficit, preserved some extent of ability for recognition via sensorimotor pathways (Sirigu et al., 1991). However, arguably, the most direct evidence for the internalized sensorimotor proposal comes from Shepard and colleagues’ studies (e.g., Shepard and Metzler, 1971; Cooper and Shepard, 1973). By using two-dimensional alphanumeric characters or two-dimensional projections of three-dimensional objects build from cubes, they assessed Reaction Times (RTs) required to determine whether two samples were identical or mirror-image version of each other. They found that RTs depended linearly on the angular disparity between the two samples to be compared. These results have been interpreted to involve a mental rotation operation whose duration depends linearly with the required rotation angle. The effect was found both with familiar shapes such as letters and digits, and with unfamiliar shapes (Cooper, 1975). For example, Shinar and Owen (1973) taught subjects multiple novel polygonal shapes at their upright orientation and let subjects recognize these shapes when presented with unfamiliar orientations. They found the time to perform this judgement was dependent on the shape’s orientation relative to the upright. In another study, Jolicoeur (1988) had subjects repeatedly name images of natural objects in different orientations. It was found that the time required to name objects was dependent on the orientation at the beginning, and the effect disappeared as subjects finished more and more repetitions of objects. The failure to observe mental-rotation effects in some experiments and the vanishing of these effects with practice can be explained by the discriminability of the stimulus (Forster et al., 1996). Forster et al. (1996) showed that mental-rotation effects can be found not only with mirror-image discrimination tasks but also using complex (polygons) as well as simple (line segments) stimuli, provided that the discrimination task is difficult enough. Mirror-image stimuli are used to eliminate all shape differences with the exception of mirror-image symmetry to make the task difficult and independent of direct strategies by comparing some specific features of the stimuli. For example, if the sample and the comparison differ from each other by the number of sharp edges they have, the observer can accomplish the task without any detailed shape comparison (hence no need for rotation) based on the number of sharp edges. Similarly, with practice, observers may discover simple local feature differences in the stimuli and base their judgments on that criterion rather than a detailed comparison via rotation. In addition to the aforementioned behavioral evidence, electrophysiological correlates of mental rotation have also been identified. Gardony et al. (2017) found multiple EEG signals in their data collected from subjects performing mental rotation tasks including sensorimotor desynchronization, parietal desynchronization, and frontal synchronization. These signatures indicated the employment of motor processing, visuospatial processing, and working memory maintenance. In another study using the images of hands as the stimuli, EEG data from sensorimotor area showed similar pattern between mental rotation task and motor imagery task (Osuagwu and Vuckovic, 2014). Another evidence supporting the involvement of motor processing during mental rotation is the strong correlation between band suppression and the reaction time of mental rotation, as suppression was suggested to correlate with motor system activation (Michel et al., 1994; Perry et al., 2010; Umilta' et al., 2012).

In this study, we investigate how geometrical factors can be used to determine the exocentric reference-frames of the object and how mental rotation is effective in learning these reference-frames with a reinforcement learning paradigm. Previous studies suggested that geometrical properties such as symmetry and elongation play an important role in the selection of intrinsic reference-frames (Rock, 1973; Marr and Nishihara, 1978; Palmer, 1983; Palmer, 1985). For example, Mou et al. (2007) investigated how layout geometry affects the selection of intrinsic reference-frame in judging the relative direction of objects within the layout. They found that subjects behaved quicker when the heading direction was parallel to the symmetrical axis of the layout. It was also demonstrated that the axis of symmetry and the axis of elongation were selected as the intrinsic orientation of the shape (Sekuler and Swimmer, 2000). These studies indicated subjects’ preference for objects presented or arranged following symmetrical or elongated manners. However, how these factors, both independently and jointly, impact the learning and development of reference-frames of unfamiliar objects has not been addressed. To answer this question, we used meaningless texture images with various dominant symmetrical and/or elongated axes as the stimuli. In each trial, we presented an image in one of multiple orientations and one of two forms (original or flipped, i.e., mirror-image of the original), and subjects were asked to report which form they observed in each trial during the experiments. Unlike conventional object recognition tasks that trained subjects with pre-selected orientations (e.g., Cooper, 1975; Tarr and Pinker, 1989; Edelman and Bulthoff, 1992; Gomez et al., 2008), we let subjects select their reference-frames for each image independently. Further, considering the selection of the relevant reference-frame may depend on cues other than the stimulus itself, such as the edges of the monitor or other references in the laboratory, we used a virtual reality (VR) headset to present our stimuli to minimize the effects of these cues. We hypothesized that subjects would perform mental rotation to transform images being presented in various orientations to learn and select reference axes for them based on their symmetry and elongation, and thus spend the shortest time on the task when the images are presented aligning such axes.

## 2 Experiment 1: symmetry and reference axis

### 2.1 Methods

#### 2.1.1 Sample size

In previous studies involving mental-rotation strategies, significant dependency of reaction time on orientation for both two-dimensional and three-dimensional stimuli have been reported with subject sizes ranging from six to nine (Shepard and Metzler, 1971; Cooper, 1975; Hock and Tromley, 1978; Matsakis et al., 1993; Leone et al., 1995; Harris and Miniussi, 2003). To determine the sample size, we ran an *a priori* power analysis using the Pingouin package in a Python environment (Vallat, 2018). In an experiment with a similar design as the present study, Tarr and Pinker (1989) reported a significant effect of orientation on reaction time with twelve subjects and four orientations based on ANOVA (*F*(3,33) = 21.05, *p* < 0.001; these results are shown on page 253). The effect-size measured by Cohen’s F in this case was 2.29 (Cohen, 2013), which was used to determine the sample size. The analysis indicated that a sample size of 5.23 participants would have a power of 95% to detect an effect of orientation on reaction time. Based on these results, we recruited subjects until seven of them (including one of the authors) successfully completed each of the three experiments in this study. To bolster further our statistical conclusions, we report and compare results from both classical ANOVA and Bayesian ANOVA for all the experiments.

#### 2.1.2 Participants

Seven students from the University of Denver, including one of the authors (DH), participated in this experiment [one female and six males; age: M(SD) = 26.57(2.61) years] and all participants had normal or corrected-to-normal vision. This experiment followed a protocol approved by the University of Denver Institutional Review Board for the Protection of Human Subjects. Each observer gave written informed-consent before the experiment.

#### 2.1.3 Stimuli and procedure

**Figure 1:**
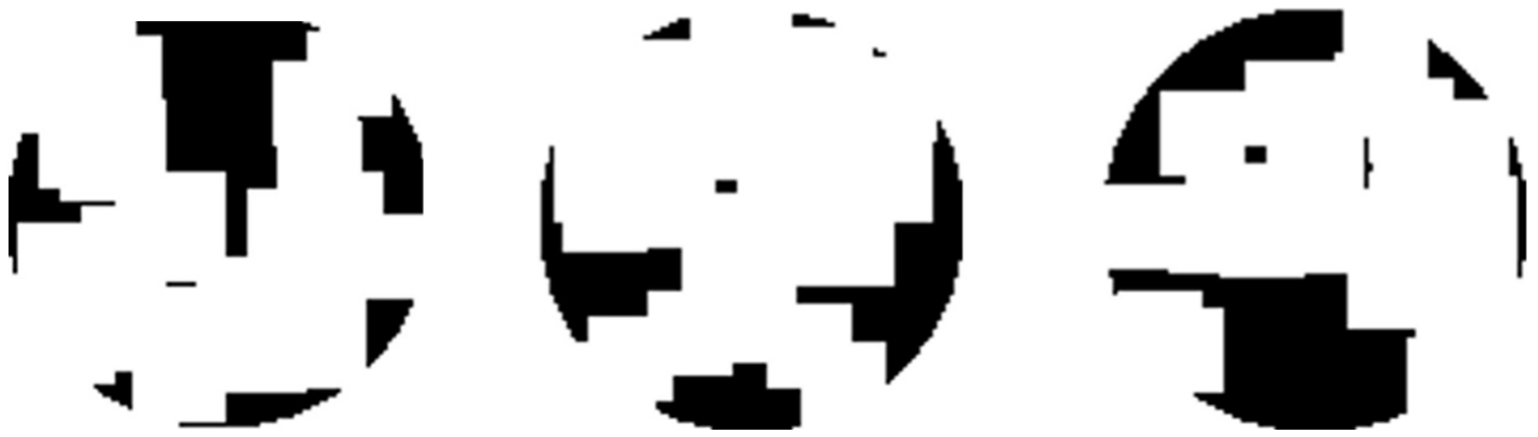
The stimuli used in the Experiment 1. The labels of these three textures are T1, T2, and T3, from the left to right.

**Figure 2:**
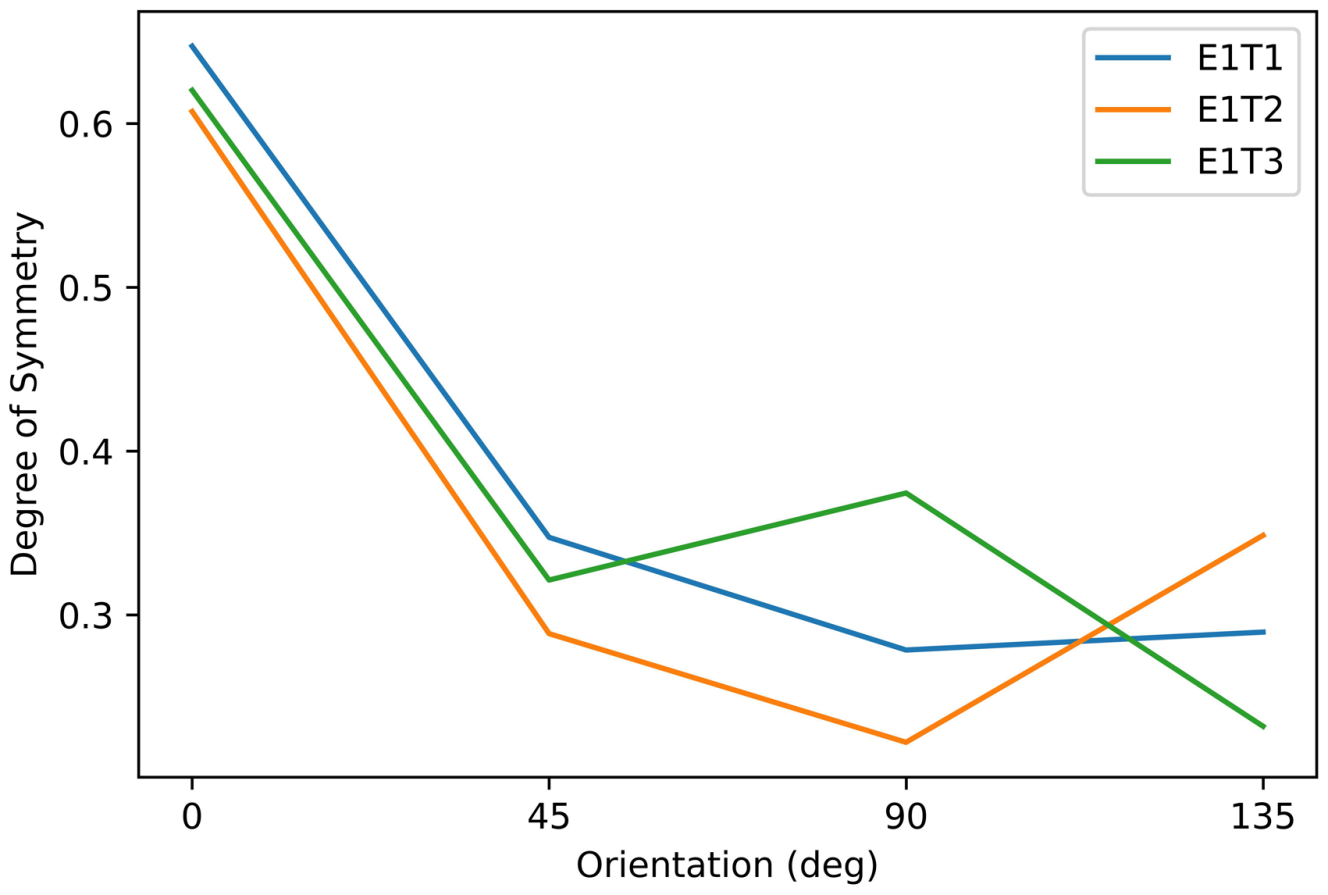
The relationship between the degree of symmetry and orientation of the stimuli in Experiment 1. Three lines correspond to the three objects, as indicated by the labels, which refer to the object labels shown in Figure 1.

Subjects observed the stimuli through an HTC VIVE VR headset (See Appendix for the calibration information of this device). They were seated in front of the headset, which was fixed on the table, with their eyes approximately 5 cm from the screens. As shown in Figure 1, the stimuli consisted of three asymmetrical black textures (T1, T2, T3). These textures were iteratively generated by randomly extracting square patches from a disk until the difference in degree of symmetry between the most symmetrical and the second most symmetrical orientations reached 0.2. Labeling the orientation of the images shown in Figure 1 to be 0°, stimuli were shown by different orientations reported in degrees measured either clockwise or counterclockwise. Although the three images are not perfectly symmetrical, the degree of symmetry along some axes is still higher than for other axes. We calculated the degree of symmetry for each stimulus along each testing axis by the proportion of overlapping areas between the two halves after folding one side to the other along the axis. The results are shown in Figure 2. As can be seen from this figure, the highest degree of symmetry is obtained for the orientation labeled 0° (i.e., the orientation at which they appear in Figure 1). As illustrated above, these textures were generated through random iterations and do not contain any semantic meanings or features other than their geometrical properties, thus we think these images pertain to the ability of generalization.

**Figure 3:**
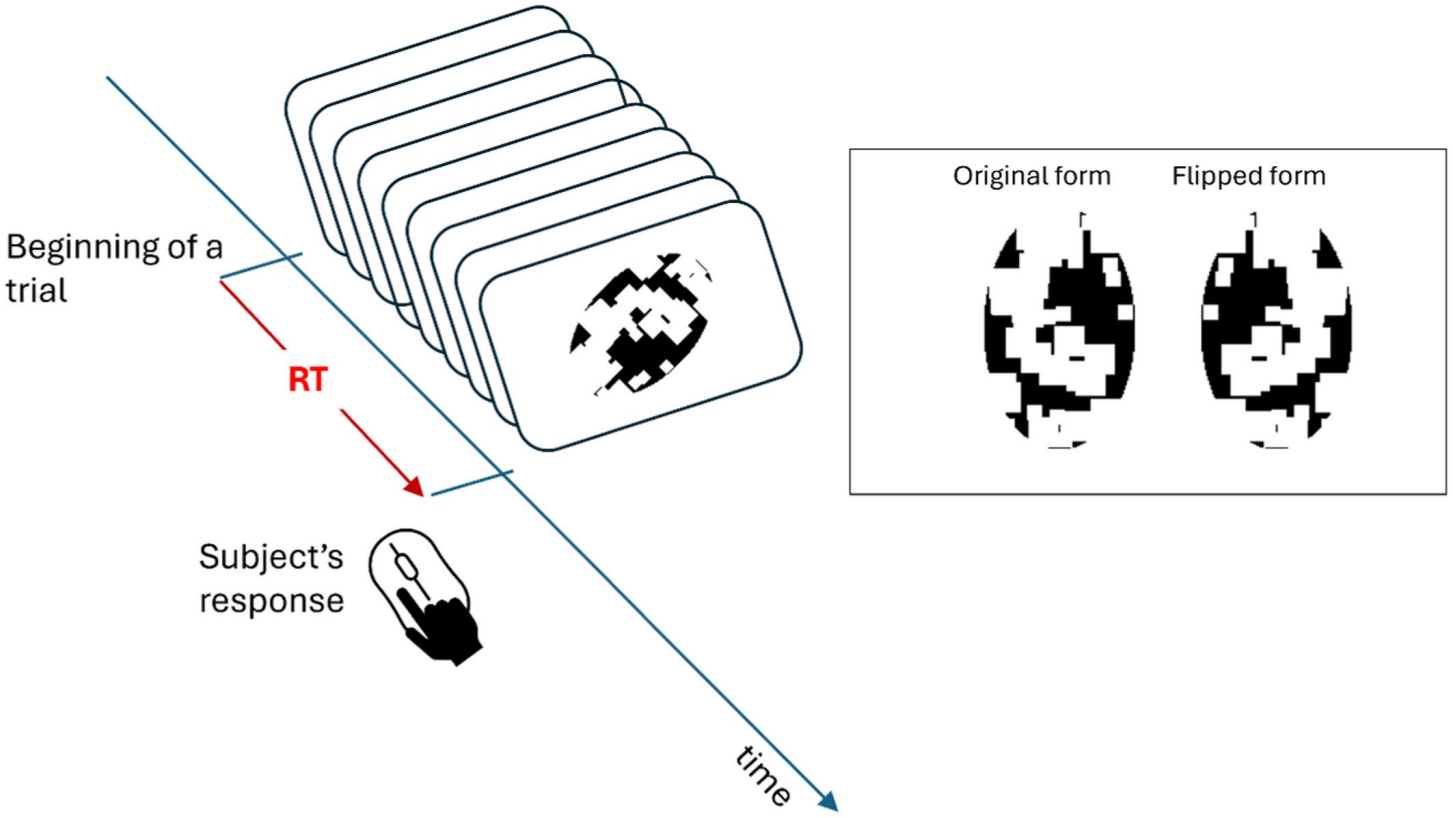
Schematic of an experimental trial. The top right panel shows an example stimulus in its “original” (on the left) or mirror-image “flipped” version (on the right). In each trial, a stimulus was presented with 50% chance either in its original or flipped version along one of the rotation angles (independent variable of the study) used in the study. After the presentation of this stimulus, the subject’s task was to indicate, by pushing one of two keys, whether the stimulus was in its original or flipped form, and the next trial was triggered at the same time. After each trial both the accuracy of the response and the Reaction Time (RT) were recorded.

In this experiment, each trial contained a stimulus (RGBA values: 0, 0, 0, 255, corresponding to $31.76\mathrm{cd}/m^{2}$ ) at the center of the visual field (RGBA values: 183, 179, 179, 255, corresponding to $107.22\mathrm{cd}/m^{2}$ ) with 50% chance either in its original or flipped version along one of the rotation angles (independent variable of the study) used in the study. The task of the observer was to report whether the stimulus was shown in its original or flipped form by pressing the left (original) or the right (flipped) key of a computer mouse. A schematic of these procedures can be found in Figure 3. Subjects were not instructed about any shape information of the stimulus at the beginning and had never observed these images before the experiment. Previous research indicates that observers accomplish this task by mentally rotating the stimulus according to an axis that enables the best contrast between the original and mirror-image flipped versions of the stimulus (Jolicoeur, 1985, 1988; Tarr and Pinker, 1989; Charles Leek and Johnston, 2006). Moreover, these papers also show that subjects’ RTs are linearly related to the amount of rotation needed for the task (Jolicoeur, 1985, 1988; Tarr and Pinker, 1989; Charles Leek and Johnston, 2006). Additionally, since subjects were naïve to the shape of each image, they had to learn to distinguish between each image’s original and flipped forms from the feedback. Therefore, a feedback beep followed wrong responses after each trial. Once the subject clicked the mouse key, the next trial followed automatically. The stimulus was presented in an orientation that was selected randomly from one of 0°, 45°, 90°, 135°, 180°, by either clockwise or counterclockwise. The distribution of orientations was uniform over these angles. In a given trial, the stimulus shown could be any one of the three shapes, five orientations, two rotation directions, and two forms. In each block, each case was shown only once, and the types of trials were selected according to a random sequence. Hence, there were 60 trials in each block. Each subject was asked to finish three blocks and was allowed to rest for a brief period between the blocks, which lasted typically less than 1 min. Since all these images were novel to subjects and only one image in single orientation was presented in each trial, subjects were able to learn and develop the reference-frames of images independently and gradually during the experiment.

Blocks with the subjects’ performance under 85% correct were excluded from the analysis. As in previous studies that used the mental rotation paradigm, in each condition of this experiment, data from all shapes and subjects were pooled together to analyze but data representing incorrect answers were excluded from the analysis (Shepard and Metzler, 1971). For each subject’s reaction time (RT) on each shape, data that were out of the range set by three times standard deviation plus/minus median or longer than 10 sec were not included in the analysis. Note that this reaction time was measured from the beginning of the stimulus onset to the time subjects pushed the mouse key to enter their response. This duration includes the time spent on subjects’ sensory and task-related processing of the stimulus, decision making, and the motor process involved in executing the response. However, except for the task-related processing, other processes are assumed to be relatively constant in duration. Therefore, we considered variations in this “reaction time” to reflect the variations in task-related processes. Moreover, since this experiment examined the effect of symmetry on the selection of a reference-frame, considering the equal level of symmetry between 0° and 180° orientations, we fitted the RT results as a function of orientation angles for each subject and each shape, by combining the orientations with equal symmetries. In practice, this was achieved by the linear regression on each subject’s reaction times to each object with respect to the orientation. We rotated the linear trend of 180°-orient reaction times to 0°-orient reaction times, which were determined by negative and positive slopes respectively, and standard errors within two times Z-score. Therefore, 0° represented the preferred most symmetrical orientation in the plots shown in the results, in which within-subjects mean and SEM are plotted.

### 2.2 Results

**Figure 4:**
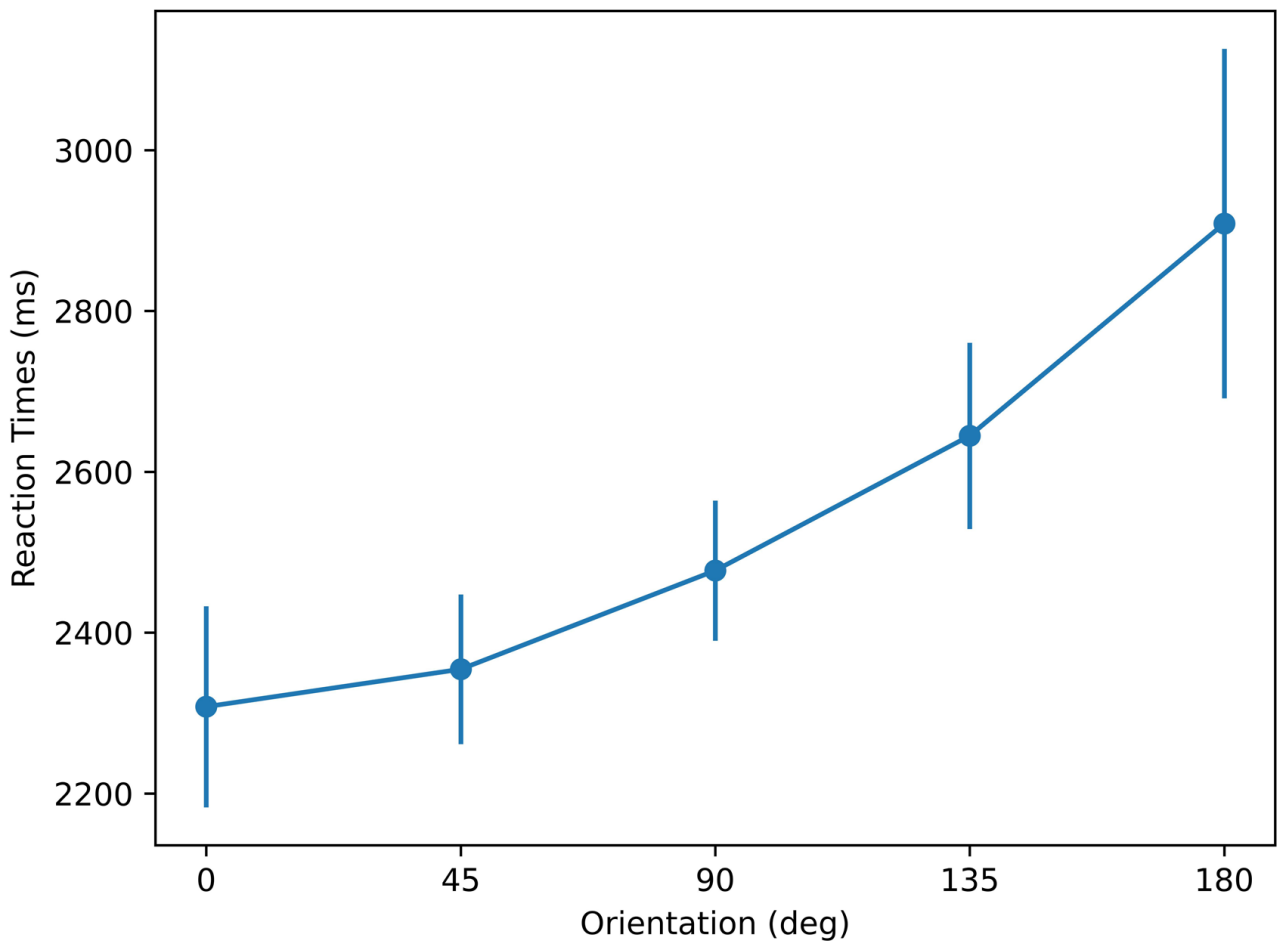
Reaction times with respect to orientation for the stimuli used in Experiment 1. Error bars represent ± 1 standard error of the mean (SEM) across subjects.

The mean [standard deviation] values of subjects’ performance in the three blocks were 83.75% [0.11], 95% [0.03], and 95.14% [0.03] respectively, and the first block of three of the subjects was excluded due to unsatisfactory performance (reflecting the learning phase, rather than the “steady-state” learned phase). As shown in Figure 4, the shortest mean RT corresponds to the orientation of 0°. The mean RT with respect to orientation profiles show linearity as reported in previous studies using the mental-rotation paradigm (Shepard and Metzler, 1971; Cooper and Shepard, 1973; Cooper, 1975), reflected by an *R*-squared value of 0.92. A repeated-measures ANOVA with orientation as the main factor showed a significant effect of orientation on RT [*F* (4, 24) = 6.5, *p* < 0.01]. Moreover, *t*-test showed the slope was significantly different from 0 [*p* < 0.01]. These are consistent with the results from a Bayesian repeated-measures ANOVA, analyzed using the software JASP (Love et al., 2019), which indicates a strong effect of orientation on RT $\left[BF_{10}=22.12\right]\text{.}$

The findings of this experiment provide support for the sensorimotor theory and highlights the role of symmetry in determining the reference axis: (i) RTs for recognition are at a minimum for the angle representing maximum symmetry, (ii) RTs follow a linear trend, supporting a mental rotation process whose duration is linearly related to the angle of orientation needed to align the stimulus with the memory prototype stored according to its reference axis.

## 3 Experiment 2: aspect ratio and reference axis

Previous research showed that aspect ratio can also play an important role on how shapes are perceived and recognized (Sekuler, 1996; Sekuler and Swimmer, 2000; Davis et al., 2003). Furthermore, elongation and orientation are ubiquitous in nature and in fact the visual system is organized to analyze orientation information through orientation columns in early cortex. Here, we tested the role of aspect ratio in determining the reference axis.

### 3.1 Methods

#### 3.1.1 Participants

Seven students from the University of Denver, including one of the authors (DH), participated in this experiment [one female and six males; age: *M* (SD) = 27 (2.73) years] and all participants had normal or corrected-to-normal vision. This experiment followed a protocol approved by the University of Denver Institutional Review Board for the Protection of Human Subjects. Each observer gave written informed consent before the experiment.

#### 3.1.2 Stimuli and procedure

**Figure 5:**
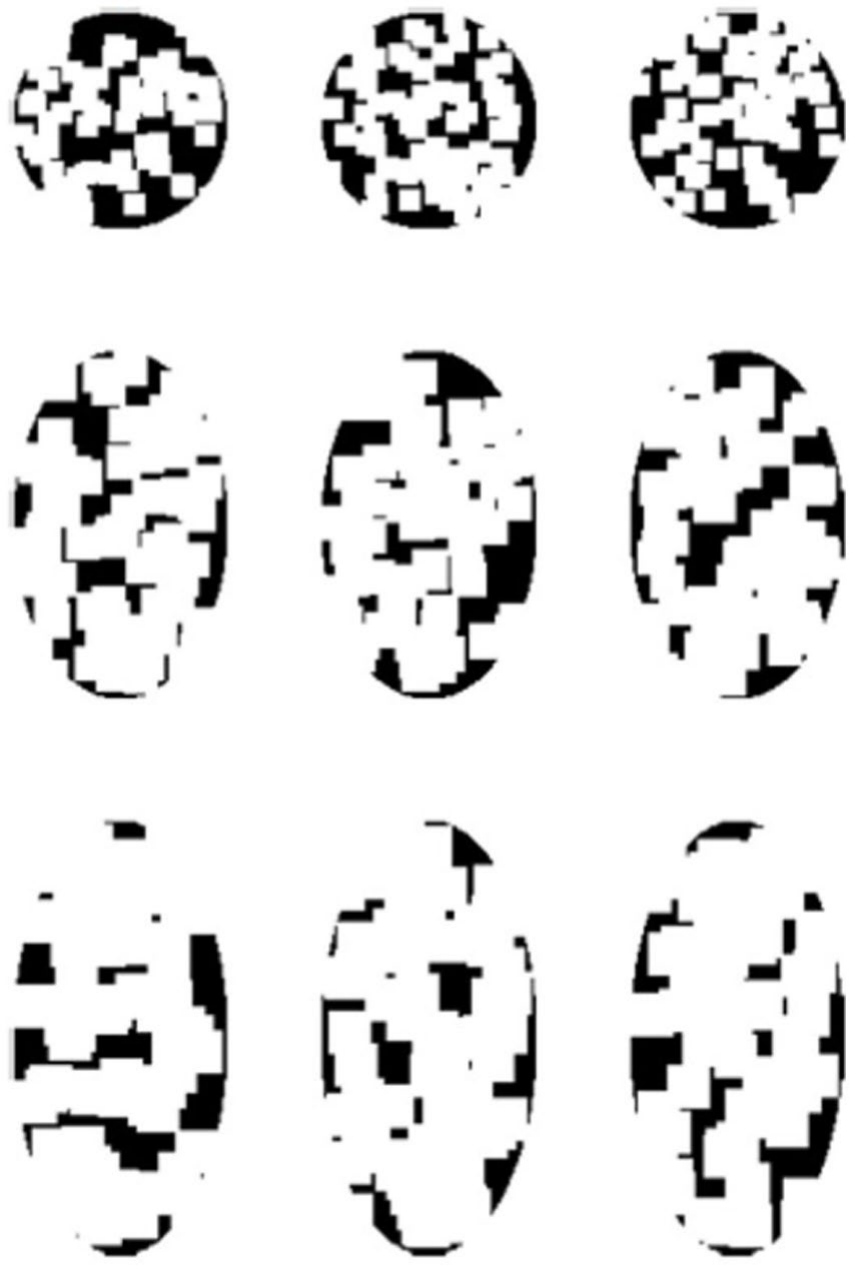
The stimuli used in the Experiment 2. The three rows from top to bottom correspond to three different aspect ratios: 1, 1.6, and 2. For each texture, the degrees of symmetry across all tested orientations are similar.

**Figure 6:**
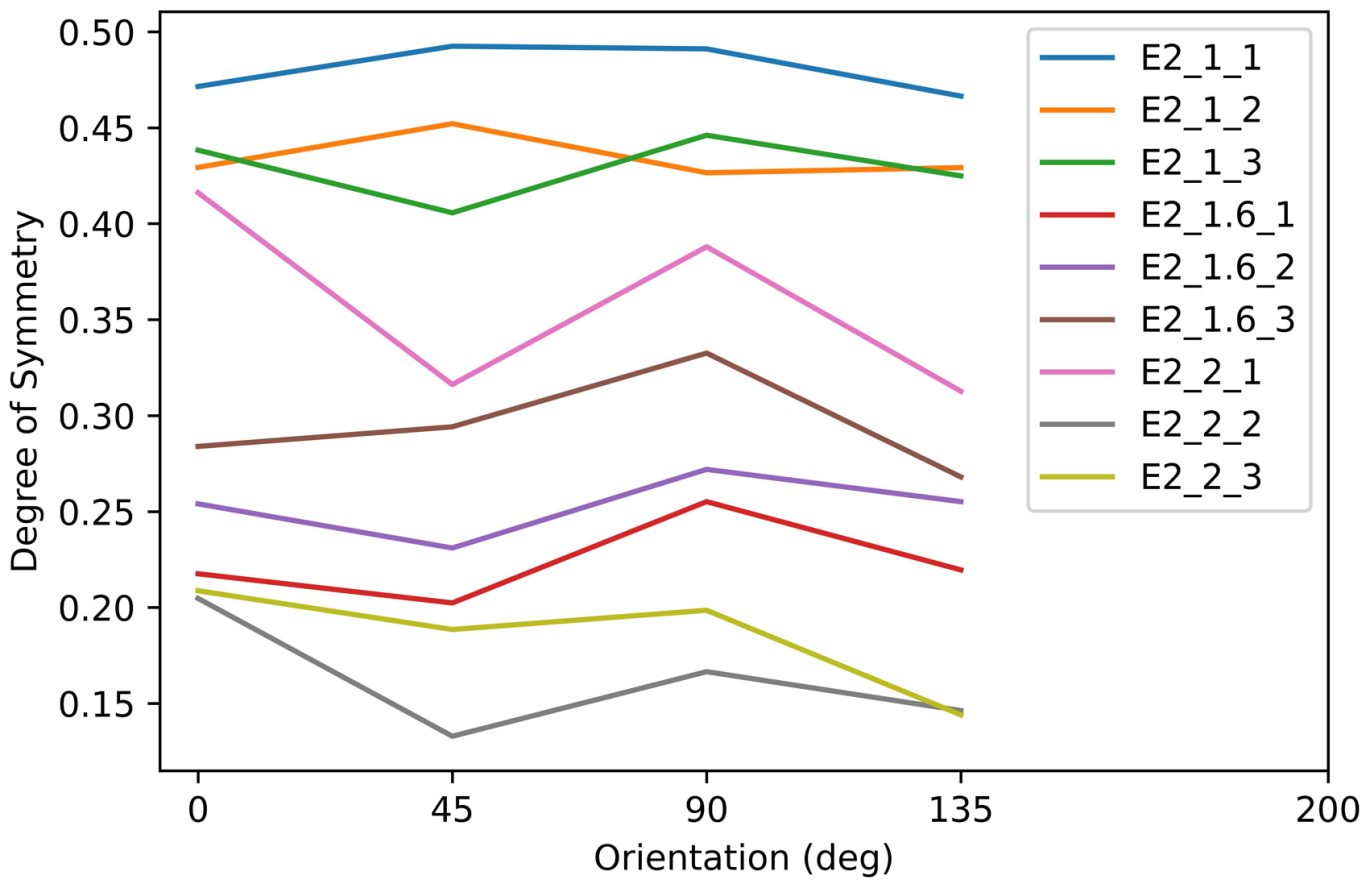
The relationship between the degree of symmetry and orientation of the stimuli in Experiment 2. Each color of line corresponds to an object in Figure 5, as indicated by the label. For example, E2_1.6_3 indicates the object with the aspect ratio of 1.6 and in the third column of the array in Figure 5.

As shown in Figure 5, there were nine textures used as the stimuli in this experiment. These textures were iteratively generated by randomly extracting square patches from a disk or an ellipse with a specific aspect-ratio defined by the length of major axis (AR = 1: 2.5 deg.; AR = 1.6: 3.16 deg.; AR = 2: 3.54 deg) divided by the length of minor axis (AR = 1: 2.5 deg.; AR = 1.6: 1.975 deg.; AR = 2: 1.77 deg). The iteration stopped once the difference between the max and min symmetry was smaller than 0.05. As we show in Figure 5, elongated along the 0° orientation, the three rows of textures are in three different aspect ratios (AR): 1:1, 1.6:1, and 2:1, and all textures have approximately equal degree of symmetry across all tested axes. The symmetry properties of these textures are plotted in Figure 6. This experiment consisted of three sessions, in which each used the three textures with three different AR from a column in Figure 6, and each session contained three blocks. The procedures and parameters of a block were exactly the same as in Experiment 1. For each subject’s reaction time (RT) on each shape, data that were out of the range set by three times standard deviation plus/minus median were not included in the analysis. Other data pre-processing and statistical analysis procedures used in Experiment 1 were also used here.

### 3.2 Results

The mean [standard deviation] values of subjects’ performance in the three blocks were 81.88% [0.13], 93.12% [0.09], and 95.72% [0.04] respectively. In all three sessions and seven subjects, with 63 blocks in total, 12 blocks were excluded from further analysis for unsatisfactory performance (<85%).

**Figure 7:**
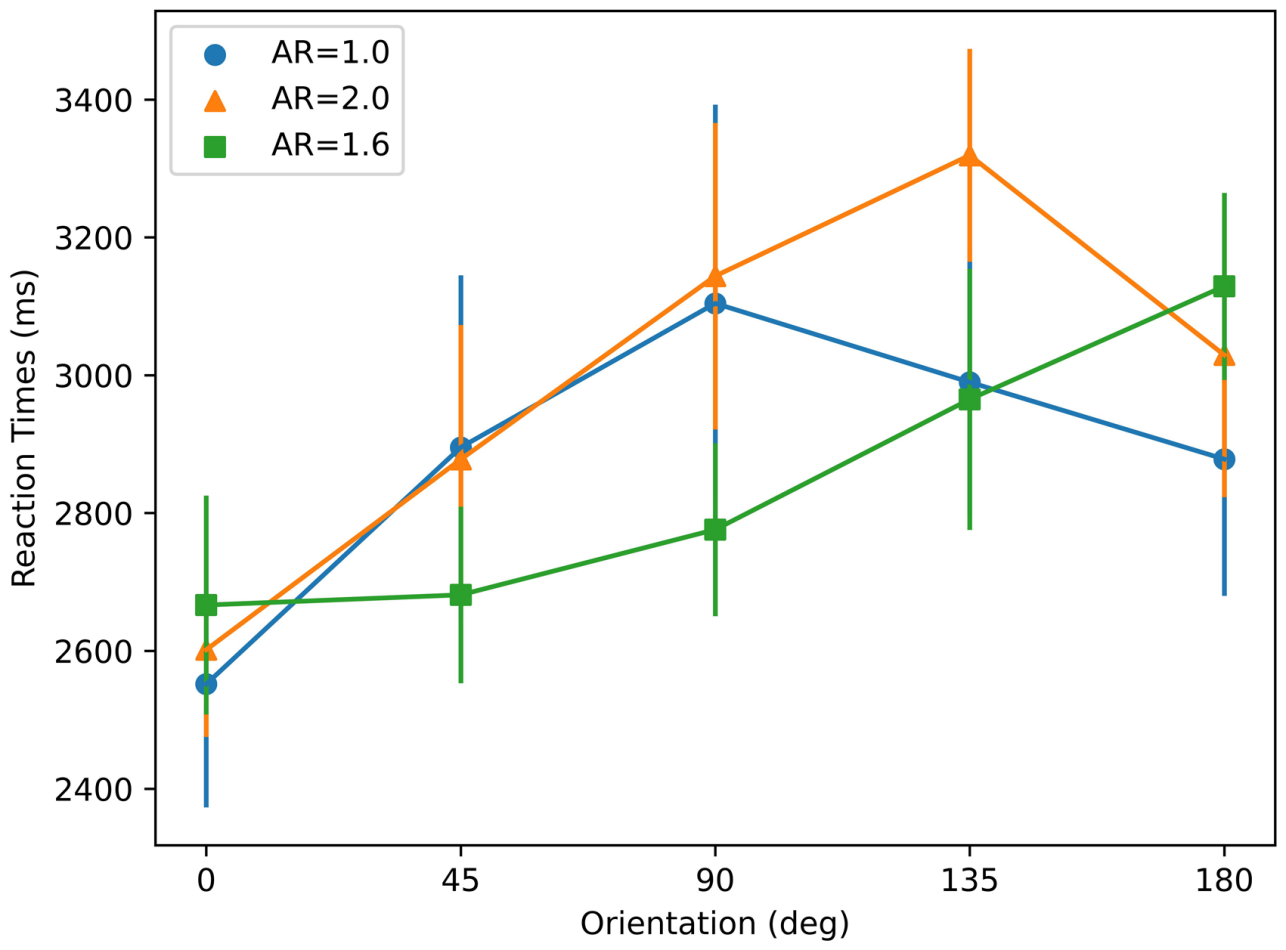
Reaction times with respect to orientation for the stimuli used in Experiment 2. Three colors correspond to three different aspect ratios (ARs). Error bars represent ±1 SEM across subjects.

A repeated-measures ANOVA with orientation and AR condition as main factors showed a significant effect of orientation on RT [*F* (4, 24) = 7.49, *p* < 0.01], and a significant interaction between AR condition and orientation on RT [*F* (8, 48) = 2.58, *p* = 0.02]. As shown in Figure 7, RTs for AR = 1.6 and AR = 2 show an increasing trend whereas RTs for AR = 1 do not, as suggested by the linear regression. A t-test showed that the slopes for these elongated AR conditions were significantly different than 0 (AR = 1.6: *p* < 0.01; AR = 2: *p* < 0.01). However, for AR = 1, t-test showed that the slope was not significantly different than 0 (*p* = 0.21). The mean RT with respect to orientation profiles for both of the two elongated AR conditions show higher linearity than the AR = 1 condition, reflected by the R-squared values: 0.32 (AR = 1), 0.92 (AR = 1.6), and 0.56 (AR = 2). These are consistent with the results from a Bayesian repeated-measures ANOVA, which indicates a strong effect of orientation for two elongated AR conditions [AR = 1.6: $BF_{10}=24.29$ ; AR = 2: $BF_{10}=22.77$ ], but indicates poor evidence for AR = 1 [ $BF_{10}=2.48$ ].

To manipulate the aspect ratio, the figure is elongated along a given axis, e.g., the circle becoming an ellipse. This geometrical transformation also creates a symmetry axis based on boundary information along the axis of elongation, thereby creating correlated aspect ratio and symmetry properties. In order to isolate aspect-ratio from symmetry, we introduced texture to the figure in a way texture did not have any preferred symmetry axis. Although this does not completely override boundary-based symmetry, it reduces its effect by making aspect-ratio more prominent than symmetry. The AR = 1 condition is a baseline control condition since it contains no dominant axis. The prediction for this case is that there will be no specific reference-axis triggered. In fact, data on the slope and the degree of linearity of the RT results for AR = 1 support this prediction. On the other hand, if a dominant component based on aspect ratio in selecting a reference axis existed, we would expect linear RTs oriented from 0° orientation.

## 4 Experiment 3: joint contributions of symmetry and aspect ratio

Previous experiments showed that both boundary and texture information can guide the selection of the reference axis for memory storage and pattern recognition. In this experiment, we studied how the reference axis is selected when boundary and texture information provide different solutions. We considered three hypotheses:

1. *Winner-take-all:* In this case, one of the two factors, symmetry or aspect ratio, dominates the selection of reference axis. This leads to a reaction time profile with respect to the angular disparity between the input orientation and the orientation of maximum symmetry or aspect ratio.
2. *Dual reference axis:* In this view, both the most symmetrical and the most elongated orientation can serve as the reference axis and remain in human memory when they are not parallel. With an input shape is to be recognized, it should be rotated to the nearest reference axis. The reaction time is then dependent on the smaller angular disparity between input orientation and two reference axes.
3. *Weighted-combination of two reference-axis candidates:* Following this view, the reference axis should be on an axis between the orientation of the maximum symmetry and another one of maximum aspect ratio.

These hypotheses can be described by the following equation:


(3) $$RT=W_{AR}\cdot k\cdot \left|\theta -\theta_{AR}^{*}\right|+W_{Sym}\cdot k\cdot \left|\theta -\theta_{Sym}^{*}\right|+T_{e}$$

where RT is the reaction time, $W_{AR}$ and $W_{Sym}$ are the steady-state weights of aspect-ratio and symmetry based reference axiss, respectively, θ is the input orientation of the shape, $\theta_{AR}^{*}$ and $\theta_{Sym}^{*}$ are the aspect-ratio and symmetry based reference axis respectively, k is the rotation speed, and $T_{e}$ is the baseline time determined by multiple factors including the encoding of shape, memory transfer, time spent on determining $W_{AR}$ and $W_{Sym}$ , preparation and execution of the motor response, etc.

The three hypotheses can then be expressed by:


(4) $$\mathrm{Winner}-\mathrm{take}-\mathrm{all}:\{\begin{array}{c} W_{K}=1,\mathrm{if}\;K\;\mathrm{dominates}\;\\ W_{K}=0,\mathrm{if}\;K^{\prime }\;\mathrm{dominates} \end{array}$$

(5) $$\mathrm{Dual}\;\mathrm{canonical}\;\mathrm{orientation}:\{\begin{array}{c} W_{K}=1,\mathrm{if}\left|\theta -\theta_{K}^{*}\right|\leq \left|\theta -\theta_{K\prime }^{*}\right|\;\\ W_{K}=0,\mathrm{if}\left|\theta -\theta_{K}^{*}\right|\geq \left|\theta -\theta_{K\prime }^{*}\right|\; \end{array}$$

(6) $$\mathrm{Weighted}-\mathrm{combination}:W_{K}=f\left(K,K^{\prime }\right)$$

where K can be either aspect ratio or symmetry and $K^{\prime }$ is the other one, $f\left(K,K^{\prime }\right)$ is a normalized function that is dependent on the magnitude of aspect ratio and symmetry.

### 4.1 Methods

#### 4.1.1 Participants

Nine students from the University of Denver, including one of the authors (DH), participated in this experiment [two females and seven males; age: *M* (SD) = 23.17 (3.67) years] and all participants had a normal or corrected-to-normal vision. This experiment followed a protocol approved by the University of Denver Institutional Review Board for the Protection of Human Subjects. Each observer gave written informed consent before the experiment. Two subjects were excluded from the analysis since they underperformed (<85%) in 10 or more blocks. Hence, data from seven subjects were included in the analysis.

#### 4.1.2 Stimuli and procedure

**Figure 8:**
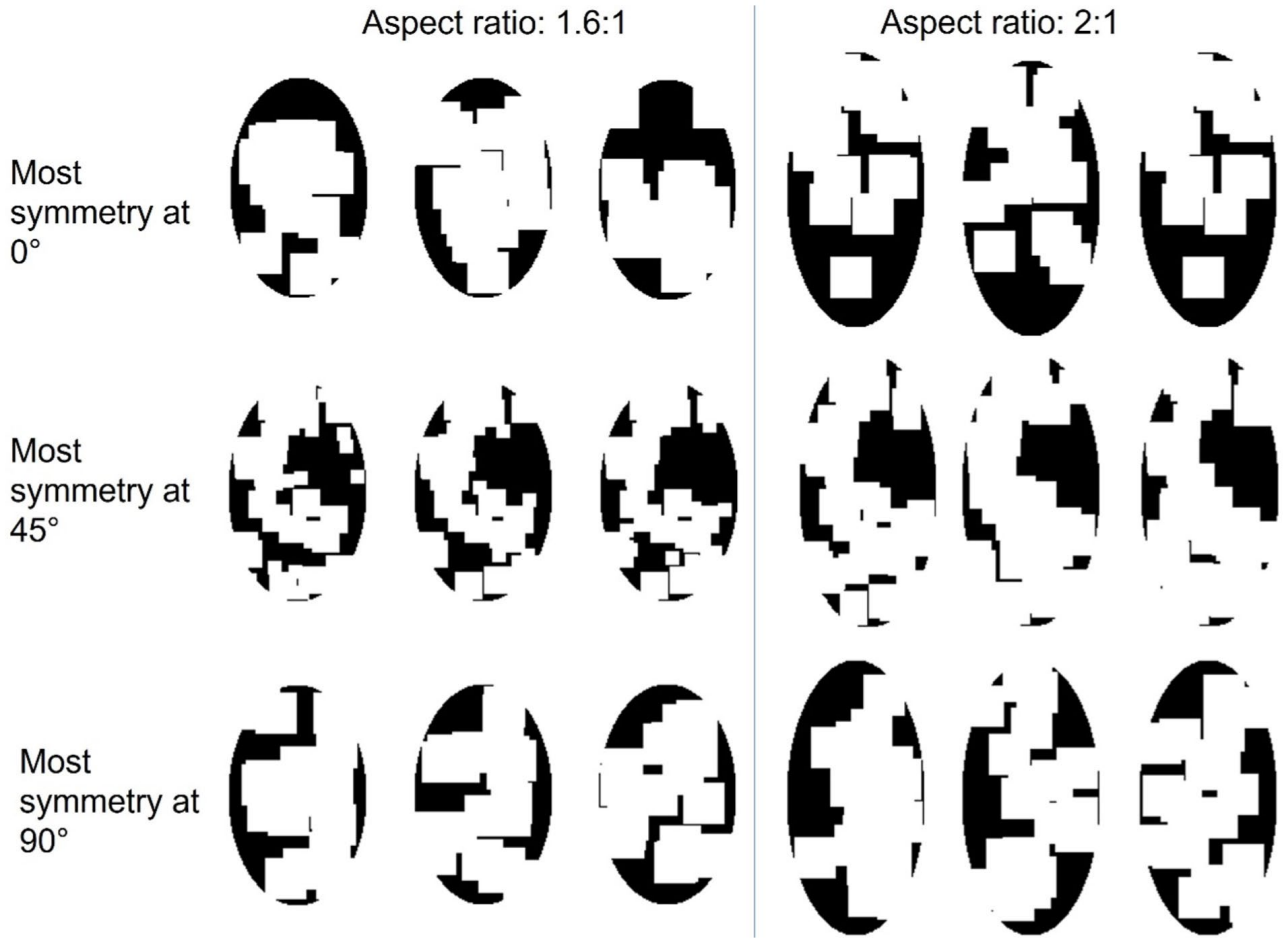
The stimuli used in Experiment 3. There are three symmetrical conditions, two aspect ratios, and three object for each symmetry-elongation combination.

**Figure 9:**
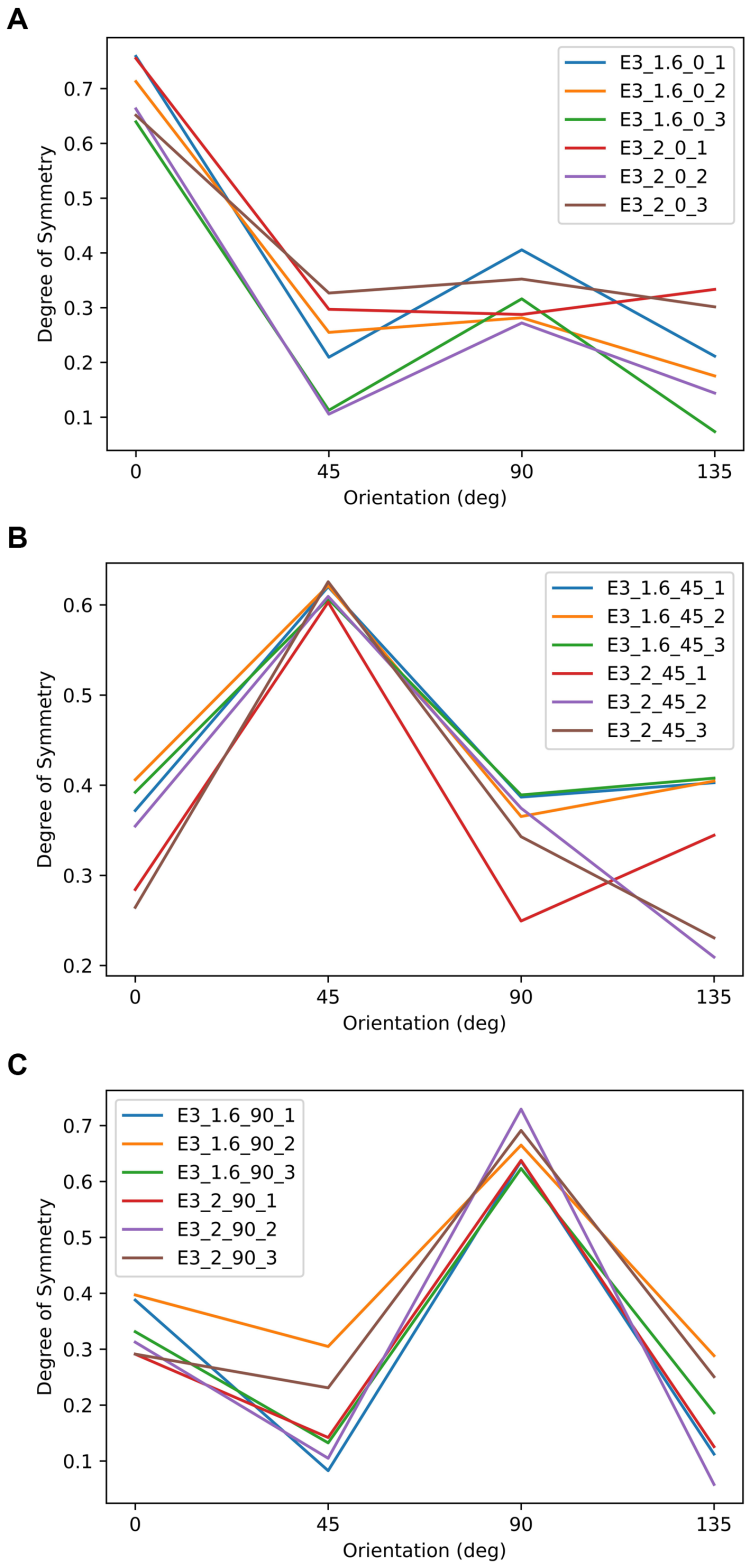
The relationship between the degree of symmetry and orientation of the stimuli in Experiment 3. These three panels correspond to the three symmetrical conditions: most symmetrical at 0°, 45°, and 90°. In each panel, each line represents to an object in Figure 8, as indicated by it label. For example, E3_2_45_1 indicates the object that is most symmetrical at 45°, has an aspect ratio of 2, and is the first item from left to right in Figure 8 under the same.

As shown in Figure 8, there were 18 textures used as the stimuli in this experiment generated in the same way as in previous experiments. The iteration stopped once the most symmetrical axis had a symmetry measure of at least 0.2 unit larger than the second most symmetrical axis. Figure 8 shows these textures in two panels, where the textures with AR of 1.6 are on the left side and those with AR of 2 are on the right side. All were elongated along 0° The three rows indicate three dominant symmetrical axes: 0°, 45°, and 90°. Therefore, the reference axis according to symmetry and according to aspect ratio could be parallel (most symmetrical at 0°), diagonal (most symmetrical at 45°), or perpendicular (most symmetrical at 90°). The symmetry properties of these textures are plotted in Figure 9.

This experiment consisted of six sessions, in which each session used the three textures with three different symmetrical properties and the same AR from a column in Figure 8. Each session contained three blocks. The procedures and parameters of a block were exactly the same as in Experiment 1 and the same data pre-processing and statistical analysis procedures as in Experiment 1 were used.

### 4.2 Results

The mean [standard deviation] values of subjects’ performance in the three blocks were 84.82% [0.12], 94.9% [0.07], and 96.53% [0.03] respectively. In all six sessions and seven subjects, 13 blocks were excluded from further analysis for unsatisfactory performance.

**Figure 10:**
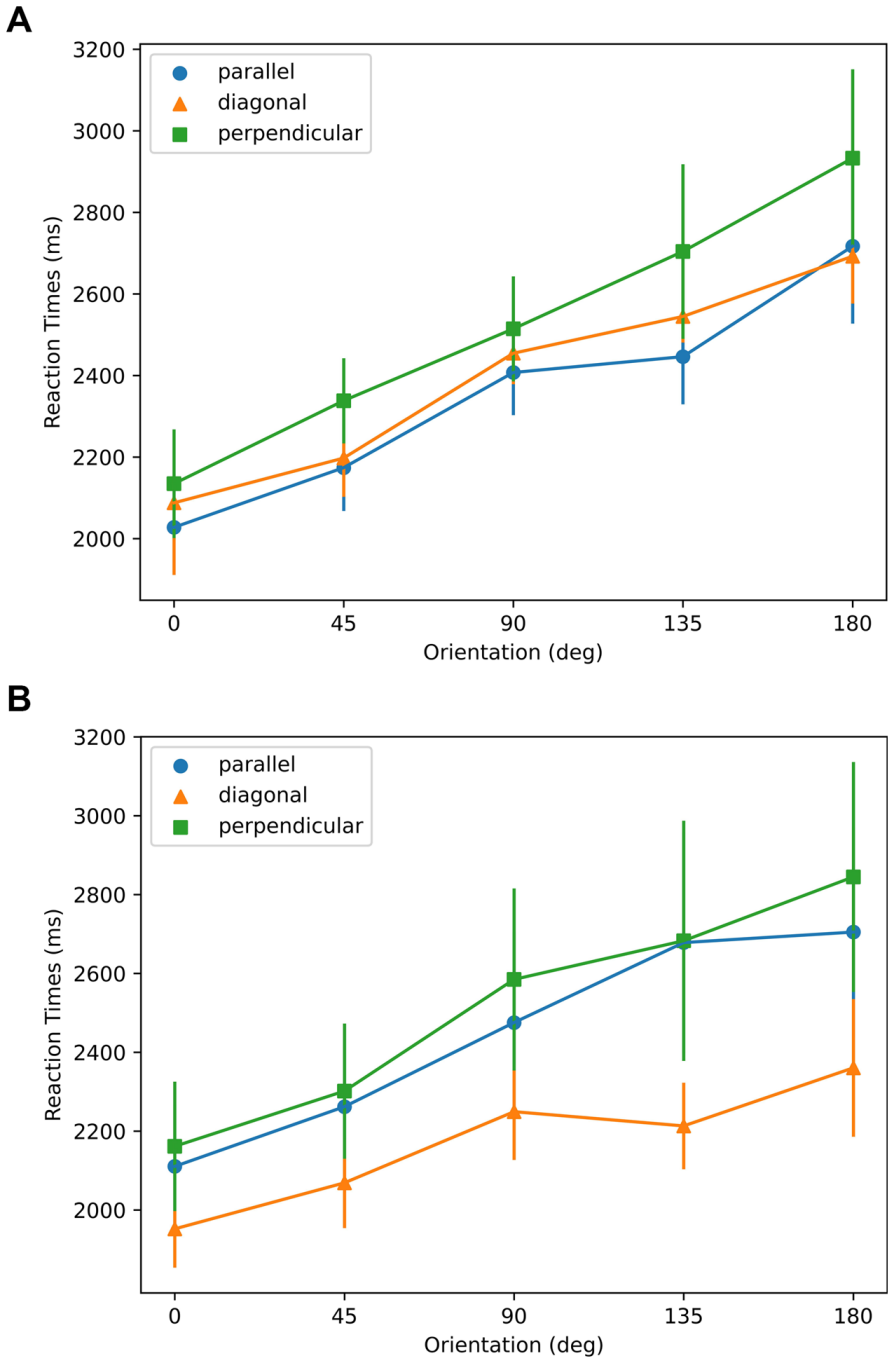
Reaction times with respect to orientation for the stimuli used in Experiment 3. **(A)** AR = 1.6; **(B)** AR = 2. Parallel, diagonal, and perpendicular conditions correspond to the stimuli in the top, middle, and bottom rows of Figure 8, respectively. Error bars represent ±1 SEM across subjects.

As shown in Figure 10, for all symmetry and aspect ratio conditions, RTs were lowest at the 0° orientation, in spite of three joint axes conditions. As shown in Figure 10 a, in the AR = 1.6 condition, linearity was found in the mean RT ~ Orientation profiles from all three symmetry-elongation conditions (parallel: $R^{2}$ =0.96; diagonal: $R^{2}$ =0.98; perpendicular: $R^{2}$ =0.99). As shown in Figure 10 b, in the AR = 2 condition, the linearity of mean RT ~ Orientation relationship reflected by R-squared values are as follows: parallel: $R^{2}$ =0.96; diagonal: $R^{2}$ =0.90; perpendicular: $R^{2}=$ 0.96. According to the t-test of linear regression, the slopes for all the conditions were significantly different than 0 with *p*-values smaller than 0.01.

A repeated-measures ANOVA with orientations, AR, and dominant symmetry orientations as main factors showed a significant effect of orientation on the RT [orientation: *F* (4, 24) = 14.15, *p* < <0.01], but did not find any other significant effect nor interactions. These are consistent with the results from a Bayesian repeated-measures ANOVA, which indicates a strong effect of orientation on RT [ $BF_{10}=3716.89$ ], but an anecdotal non-effect of condition [ $BF_{10}=0.98$ ], and a moderate non-effect of the interaction between orientation and condition [ $BF_{10}=0.36$ ].

The data support the sensorimotor theory as the mental rotation strategy was used, indicated by the linear relation between the RTs and the orientations. The 0 deg. orientation in Figure 10 corresponds to the optimal orientation according to aspect ratio. The optimal orientations according to symmetry are 0, 45, and 90 deg. for parallel, diagonal, and perpendicular conditions, respectively. The existence of a minimum at 0 deg. in the data support the Winner-take-all hypothesis, and the aspect ratio as the dominant feature compared to the symmetry.

In the case of winner-take-all hypothesis, we were expecting RTs to be lowest in the congruent parallel condition, followed by the incongruent diagonal and perpendicular conditions. This is because, the smaller the difference between the two optimal orientations, the faster we expected the competition between the two factors to settle. We observe this tendency in the AR = 1.6 condition but not in the AR = 2.0 condition. One possible reason could be the feature of shapes. When AR was 2, to meet the symmetrical properties along each axis, the texture of shapes was unbalanced comparing the left and right sides, as shown in Figure 8. With one side more filled than the other, the task could become easier by using this cue. However, let us note that the effect of condition (parallel, diagonal, perpendicular) was not significant in our data and these observations are not conclusive. An important point in considering how different stimulus properties are combined concerns their salience. It is possible, for example, that aspect ratio is much more salient than symmetry in our stimuli. These two stimulus dimensions are categorically different and have different metrics. Thus, “equating” their strengths cannot be based on the stimulus properties alone, but it requires some behavioral measures assumed to equate them according to their internal representations. An example would be to vary the stimulus to match the reaction times required to detect the relevant axes based on aspect ratio vs. on symmetry. In our study, we examined the joint effects of these factors while keeping our stimuli consistent with the previous experiments. Future studies can adopt the aforementioned strategy for equating these two factors. The reference-frame used is related to the task in the sense that the selected axis provides a better comparison and contrast between the original stimulus and its mirror-image flipped version. Although we did not directly assess whether and how much subjects can exert conscious control in the selection of the reference-frame, previous studies indicate that subjects may have limited direct conscious control in the selection process. For example, when we observe a stationary object during voluntary smooth-pursuit eye-movements, the representations of this object on the retina and on retinotopic areas undergo motion in the opposite direction to the eye movements. Yet, we perceive this object as stationary, i.e., according to a spatiotopic reference-frame and we cannot voluntarily switch to a retinotopic reference frame. On the other hand, if we move *passively* one eye by pushing it gently with our finger (with the other eye covered), we observe retinotopic motion since the brain does not have afferent motor-control signals for compensation. In this case too it is not possible to switch voluntarily to another reference-frame: We cannot voluntarily perceive the object stationary according to a spatiotopic reference-frame. The way spatiotopic and retinotopic reference-frames are selected depend on stimulus properties such as perceptual groups (e.g., Öğmen et al., 2006) and set-size (Huynh et al., 2017) as well as relevant internal signals available to the brain (He and Öğmen, 2023).

## 5 General discussion and conclusion

### 5.1 Roles of symmetry and elongation in developing reference-frames

Our study was designed to eliminate extraneous environmental (by using VR) and pre-learned (by using reinforcement learning) reference-frames. In all the conditions, on average, subjects spent shortest time on recognizing objects in those orientations that were salient according to the factors we studied, i.e., symmetry and elongation. In Experiments 1 and 2, our data indicate that subjects selected either the most symmetrical or the most elongated orientation as the reference axis when these factors were present in isolation. Moreover, our data suggest a Winner-Take-All process when the two factors were simultaneously present. In Experiment 3, the shortest reaction times correspond to the elongated orientation regardless of different symmetrical axes. Previous studies provided evidence for the role of symmetry and elongation in selecting a reference frame for different objects (Rock, 1973; Marr and Nishihara, 1978; Palmer, 1983; Mou et al., 2007). For example, Sekuler and Swimmer (2000) conducted an experiment and let the subject determine the primary axis of shapes with different symmetrical and elongation axes. They found that both the axis of symmetry and the axis of elongation were sufficient for deriving the primary axis and these two factors affected each other. However, they did not address the question of how these axes are developed during the learning process when images are presented in various orientations. The results of our experiments show that both symmetry and elongation can evoke the development of reference-frames and support the sensorimotor theory of learning as our data reflect a mental rotation strategy based on the linear reaction time profile with respect to the orientation.

### 5.2 Canonical forms and object recognition

Recognizing a previously learned object requires that we match the current appearance of the object (stimulus) with the memory representations of candidate objects and determine the best match. This task is complicated because objects do not have unique appearances: As the relative position of the object with respect to the observer changes (perspective views), the appearance of the object can undergo drastic changes. Several theories have been formulated to explain how the brain accomplishes this “invariant object recognition” task (reviews: Logothetis and Sheinberg, 1996; Riesenhuber and Poggio, 2000; DiCarlo et al., 2012; Gauthier and Tarr, 2016).

According to “invariant feature/relation” approaches, this problem can be by-passed all together by using memory representations that are invariant to perspective views (Pinker, 1984; Palmer, 1999). In these theories, an object is described by a collection of low-level features such as angles, curves (Sutherland, 1968; Barlow et al., 1972), and/or higher-level structural characteristics such as component parts and their relations (e.g., Palmer, 1977; Biederman, 1987), which are independent of viewpoints. The stimulus-memory comparison takes place by matching these invariant feature/relation-based structural descriptions. For example, an edge length or a vertex angle can be compared directly regardless of the orientation of the stimulus and the memory item, as long as matching edges and vertices between the stimulus and memory representation are found. A horse can be recognized independent of its orientation based on the relations of the parts or components (head, neck, torso, legs, tail, etc.; Marr and Nishihara, 1978; Biederman, 1987).

In contrast to invariant feature/relation approaches, several theories proposed mechanisms whereby view-variant representations are combined or actively transformed to accomplish object recognition. The “perspective-storage theory” proposes that, as the subject experiences different views/perspectives of the same item, each view/perspective is stored as is under the same label (e.g., my friend Jane). This theory follows the behavioristic approach where stimuli and responses are associated with each other as they appear in the environment. For example, if we see five different perspectives of a given face and each is presented together with a name, those perspective views will be associated with that particular name. As in associative learning, when the subject experiences in the future one of the stored views, it will generate the associated response, such as the name of the person. In this theory, the internal representations consist of a set of stimuli with an associated label. In classical conditioning, the object and the label may be occurring in close spatiotemporal proximity (e.g., while an object is shown and its label/name is verbalized) and in reinforcement learning, the observer’s response is either positively or negatively reinforced until the correct response is found. In addition to behaviorism, this approach is also used extensively in artificial neural networks from early versions (Rosenblatt, 1958) to later incarnations (e.g., Fukushima (1975); Riesenhuber and Poggio (1999); Krizhevsky et al. (2017)). These are hierarchical feed-forward models where each layer filters its input and sends the filtered information to the next layer via simple nonlinearities. In these networks, memory is implicit in that it consists of distributed values of synaptic connections across the network. The “generalization,” i.e., the association of the label to novel perspectives occur via some interpolation process using similar perspectives that are already stored (Edelman and Bulthoff, 1992). In other words, memory representations and storage in these models are “passive processes” in the sense that there is no active internal structuring or manipulation of the stimuli during storage and recall.

Whereas invariant feature/relation approaches deal with variances by using invariant features, an alternative approach is to cancel environmental variations by applying inverse transforms. Hence, theories that use this approach posit an active internal structuring during storage and/or recall to compensate for the effects of environmental variances. For example, according to the alignment theory (Ullman, 1989), anchor points are used to align the stimulus with the memory (internal model) by using transformations such as scaling and rotation, a process called “normalization.” The “canonical-representation theories” (Palmer et al., 1981) use similar alignment approaches but posit that, the memory storage is not arbitrary, but follows a canonical scheme: When a stimulus appears, it is not stored as is. Instead, a canonical representation is chosen and this canonical form is stored in memory. For example, the canonical form for objects can be according to the symmetry axis for symmetric objects. To carry out the comparison between a stimulus and the memory representation, the input shape is converted to the canonical orientation through mental rotation. Several studies provided evidence against invariant features/relations theories (Tarr et al., 1998; Kourtzi and Shiffrar, 1999; Fang and He, 2005), however, tests of the other theories have been equivocal (Vanrie et al., 2001; Willems and Wagemans, 2001; Ratan Murty and Arun, 2015; Tarr and Hayward, 2017). Note that these theories are not mutually exclusive and there are also hybrid versions: For example, Tarr and Pinker (1989) proposed that we store a small set of orientation-specific representations, as in the perspective storage theory, and the input shape is transformed to match the closest one, as in the canonical storage theory.

The structural-description theory predicts that observers’ performance should be independent of the viewing rotation-angle because its coding is inherently independent of rotational changes. The perspective-storage theory predicts that observers’ performance will vary according to the statistics of perspective views; in other words, those views experienced more often should lead to better recognition. Whereas it is difficult to determine the viewing statistics of familiar objects for each and every rotation angle, one can control these statistics by using novel objects in a laboratory environment. If novel objects’ presentation follows a uniform distribution in terms of orientation angle, then the perspective-storage theory predicts the same performance independent of rotation angle. The canonical sensorimotor theory, however, predicts that performance will be best for the canonical orientation and will degrade monotonically with the difference between canonical orientation and the viewing orientation. Results of our experiments indicated that geometrical factors evoked canonical forms, as determined by the reference axes for each image through sensorimotor transformations, in memory storage during object recognition.

Some previous studies claimed that mental rotation is used only when the task is to determine the handedness and irrelevant to the cognitive processing the object recognition (Corballis et al., 1978; Hinton and Parsons, 1981). Our results, along with other studies (Jolicoeur, 1985; Tarr and Pinker, 1989; Charles Leek and Johnston, 2006), provide evidence against this claim: linearity in reaction time is *not* observed when the objects did not have any reference-frame affecting factors in Experiment 2. With same task as other conditions, the shapes in the top row of Figure 5 did not generate a linear trend in the reaction times. Importantly, in this condition, the mean reaction-time profile with respect to the orientation was not a flat curve. Therefore, the non-linearity is considered to be caused by the diversity of canonical orientations across subjects and shapes since there were no salient canonical indicators. This points out that the canonical orientation was still used with textures without any geometrically solid axis. From a more general perspective, part of the disagreement stems from the interpretation of the term “object recognition.” There is no universal agreement for the meaning of object recognition. It has been defined as “*the ability to recognize a previously experienced object as familiar*” (Weiss and Disterhoft, 2005) as well as “*to determine the identity or category of an object in a visual scene from the retinal input*.” (Mozer, 2001). Note that the determination of identity vs. category often entails different levels of difficulty. For example, it may be relatively easy to categorize an animal as a horse vs. dog vs. butterfly. On the other hand, it may be more difficult to identify a familiar horse among several other horses. As we discussed in the Introduction section, Förster et al. showed that mental-rotation effects can be observed with mirror-image stimuli as well as simple (line segments) and complex (polygons) geometric stimuli, provided that the discrimination task is difficult enough. The goal of using mirror-image stimuli is to increase difficulty by eliminating *all* shape differences with the exception of mirror-image symmetry. This approach also makes the task independent of direct strategies by comparing some specific features of the stimuli.

### 5.3 Conclusion

To conclude, our results provide evidence for reference axes determined by symmetry and elongation and support the sensorimotor theory for learning and developing reference-frames, as well as the canonical-form theory for object recognition. However, as we mentioned in the Introduction section, this does not rule out strategies that can be driven by environmental cues. Our goal in this study was to focus on figural cues (symmetry and elongation) in isolation. Future studies can examine the combination of figural and environmental cues with a method to match feature saliences and assess whether the Winner-Take-All rule also holds for those combinations.

## Data availability statement

The raw data supporting the conclusions of this article will be made available by the authors, without undue reservation.

## Ethics statement

The studies involving humans were approved by the University of Denver Institutional Review Board for the Protection of Human Subjects. The studies were conducted in accordance with the local legislation and institutional requirements. The participants provided their written informed consent to participate in this study.

## Author contributions

DH: Conceptualization, Data curation, Formal analysis, Investigation, Methodology, Software, Validation, Visualization, Writing – original draft, Writing – review & editing. HO: Conceptualization, Investigation, Methodology, Supervision, Writing – original draft, Writing – review & editing.
