# Supplementary material for: The roles of symmetry and elongation in developing reference frames
Source: Front Psychol. 2024 Jul 1;15:1402156. doi: 10.3389/fpsyg.2024.1402156 (PMC11249022; doi:10.3389/fpsyg.2024.1402156)
Supplement: Supplementary file 1 [file Presentation_1.pdf]

Appendix-Equipment & Calibration.

In an experiment conducted on a traditional monitor placed in an experimental room, subjects are exposed not only to the experimental stimuli but also to various other geometric cues that can serve as a reference-frame. For example, the rectangular shape of the display monitor can serve as a reference-frame. To avoid such cues, we presented our stimulus using an HTC VIVE VR headset released in 2017.

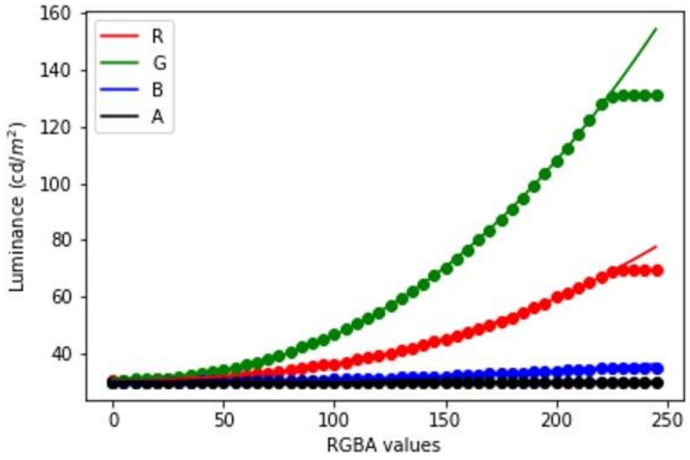

Figure A: Gamma fitting results of VR headset screen's luminance with respect to the RGBA value inputs in the Unity3D under the experimental condition.

The resolution of each of two screens in the VR headset was 1200×1080 with a 90 Hz refresh rate. The color display of the device was controlled by the development environment Unity3D using RGBA values. Since the left and right displays of the VR headset have the same parameters, we calibrated the color display by measuring the luminance of the left display with a Minolta LS-110 luminance meter while adjusting the RGBA input from Unity3D under our experimental settings. These four channels represent red, green, blue, and alpha respectively, in which alpha indicates the degree of transparency. First, we tested each color-channel separately, i.e., by varying its inputs from 0 to 255, with a sampling interval of 5, while keeping all other channels at 0. The relations are shown in Figure A. We found the minimum luminance of all four channels to be approximately $30cd/m^2$. The maximum luminance of individual RGBA channels were 75, 154, 36, and $30cd/m^2$, respectively. We fitted Gamma functions to the data and obtained the following:

$$\begin{cases} LumR = 8.47 \times 10^{-5} \times R^{2.4} + 30.87 \\ LumG = 3.43 \times 10^{-4} \times G^{2.3} + 31.13 \\ LumB = 1.59 \times 10^{-5} \times B^{2.3} + 30.14 \end{cases} \quad (1)$$

, where $LumR$, $LumG$, and $LumB$ represent luminance produced by red, green, and blue channels, respectively; R, G, and B correspond to the input values in red, green, and blue channels of Unity3D's color setting.

The RMSE of these fits were 1.54, 4.55, and 0.27 cd/m^2. As for the A channel, the luminance doesn't change with respect to the A value (t=3.07×10^{-8}, p = 0.999). We then tested the overall luminance, Lum, in relation to the RGB values and obtained the following calibration function:

$$Lum = 0.97LumR + 0.99LumG + 0.73LumB - 51 \quad (2)$$
